# Existence and distribution of the microbiome in tumour tissues of children with hepatoblastoma

**DOI:** 10.1016/j.heliyon.2024.e39547

**Published:** 2024-10-18

**Authors:** Jinghua Cui, Xiaoran Li, Qun Zhang, Bing Du, Zanbo Ding, Chao Yan, Guanhua Xue, Lin Gan, Junxia Feng, Zheng Fan, Ziying Xu, Zihui Yu, Tongtong Fu, Yanling Feng, Hanqing Zhao, Yiming Kong, Xiaohu Cui, Ziyan Tian, Quanda Liu, Jing Yuan

**Affiliations:** a Capital Institute of Pediatrics, Beijing, 100020, China; b Postgraduate Base of the PLA Rocket Force Medical Center, Jinzhou Medical University, Jinzhou, 121001, Liaoning Province, China; c Department of Hematology and Oncology, 155th Hospital of Kaifeng, Kaifeng, 475003, Henan Province, China; d Beijing Ditan Hospital, Capital Medical University, Beijing, 100015, China; e Department of General Surgery, Guang'an Men Hospital, China Academy of Chinese Medical Sciences, Beijing, 100053, China

**Keywords:** Hepatoblastoma, Microbiome, Tumour microenvironment, Children

## Abstract

Cancer microbiota have recently been demonstrated in several cancer types. The microbiome enhances inflammation in the cancer microenvironment and affects the disease pathology by regulating tumourigenesis, cancer progression, and chemotherapy resistance. Hepatoblastoma (HB), the most common childhood malignant tumour, is a malignant embryonic tumour. However, the pathogenesis and molecular basis of HB remain poorly understood. In this study, to explore the existence and distribution of the microbiome in tumour tissues and adjacent non-tumour tissues of children with HB, we mainly performed 16S rDNA sequencing, and the results showed that the diversity and abundance of the microbiome in children with HB were significantly different between HB tumours and adjacent non-tumour tissues (*p* < 0.01). At the phylum level, the dominant microbiome in the tumour tissues were Proteobacteria, Bacteroidetes, and Firmicutes. At the genus level, *Ruminococcus* was more abundant in HB tumours than in the adjacent non-tumour tissues. Simultaneously, the abundances of *Bacteroides*, *Parabacteroides*, *Lachnospiracea*-NK4A136, and *Alistipes* in HB tumours were lower than those in the adjacent non-tumour tissues. In addition, *Romboutsia* strongly correlated with alpha-fetoprotein, an important indicator of HB. *Sphingomonas* was abundant in primary HB tumours, whereas *Oscillibacter* and *Pandoraea* were abundant in metastatic HB tumours. However, whether these bacteria are associated with HB needs further evaluation. Therefore, we identified the microbiome that correlated with the occurrence and development of HB. *Ruminococcus* and *Romboutsia* were identified as potential bacterial markers of HB tumours. To conclude, we found that HB also contains cancer microbiome, and it is necessary to shed light on the microbiome characteristics of HB in the future.

## 1 Introduction

Hepatoblastoma (HB) is the most common malignant childhood tumour. It results from the abnormal differentiation of epithelial hepatocyte precursors during embryonic development [1]. Its incidence is 1.2–1.5/10^6^ per year, and mostly occurs in children under 3 years of age [2]. The onset of HB is insidious; most cases are sporadic and usually lack typical clinical features, with an abdominal mass as the main clinical manifestation. Premature birth, very low birth weight, and certain congenital disorders such as familial adenomatous polyposis are associated with an increased risk of HB [3]. With advances in surgical treatment combined with chemotherapy, the 5-year overall survival (OS) of patients with HB has increased significantly from approximately 30 % (30 years ago) to 80 % (presently) [4]. A Japanese study in 2020 showed that the 5-year OS rate had increased to 89.9 % [5]. However, the prognosis of patients with recurrent or metastatic HB that cannot be resected is still unclear [6,7]. Similar to those of many childhood tumours, the pathogenesis and molecular basis of HB are poorly understood. Therefore, exploring the pathogenesis of HB is of great clinical significance for developing new treatment strategies and reducing the risk of death from this condition.

Bacterial colonisation, known as the cancer microbiome, has recently been demonstrated in several cancer types, including colorectal and gastric cancers [8,9], and in cancers of sterile tissues, such as breast ductal adenocarcinoma, pancreatic ductal adenocarcinoma, and liver cancer [10,11]. The cancer microbiome affects the cancer pathology by regulating tumourigenesis, cancer progression, and chemotherapy resistance [12,13]. For example, *Clostridium nucleobacter* found in colorectal cancer tumour tissues, can increase pro-inflammatory cytokine levels in rodent models and patients with colorectal cancer [14]. These studies suggested that the cancer microbiome enhances inflammation in the cancer microenvironment. Most cancer microbiota belong to Proteobacteria, Firmicutes, and Bacteroidetes, suggesting that their major source is gut microbiota [15]. Given the anatomical connection of the liver to the intestine via the portal vein and the transfer of intestinal bacteria to the liver in patients with chronic liver disease [16], we speculated that HB may also exhibit cancer microbiota. The current understanding of the impact of microbiota on human health and tumourigenesis raises the question, “Do microorganisms play a role in sporadic HB of an unknown cause”?

Owing to the diversity of microbial communities among individuals, we believe that matching tissues from the same individual (tumour and adjacent non-tumour tissues) would provide the best comparison of microbial communities. Therefore, in this study, we selected tumour tissues from patients with HB treated surgically in the Hepatobiliary Surgery Department of Rocket Force Special Medical Center from June 2020 to June 2021 as test samples and adjacent non-tumour tissues as controls. We assessed the presence of bacteria in the tumour tissues of children with HB and whether there were differences in the diversity and abundance of the microbiome. This information was further used to determine the relationship between HB occurrence and development in children and the microbiome.

## 2 Methods

### 2.1 Patient selection

In total, 16 tumour tissues from patients with HB who underwent surgical resection in the Department of Hepatobiliary Surgery at the Rocket Army Special Medical Center between June 2020 and June 2021 were selected, and the adjacent non-tumour tissues from the same patients were used as controls. This study was approved by the Ethics Committee of the Rocket Force Special Medical Center (KY2024040) and adhered to the ethical principles of the Declaration of Helsinki. Written informed consent was obtained from the children's families before surgery. Patient information and clinical indicators were collected from progress notes. Sensitive patient information was anonymised and identified prior to analysis.

### 2.2 Real-time quantitative polymerase chain reaction

Real-time quantitative PCR was used to determine the total number of bacteria in liver tissues. The upstream primers (5ʹ-CCTACGGGNGGCWGCAG-3ʹ) and downstream primers (5ʹ-GACTACHVGGGTATCTAATCC-3ʹ) were used, and the reaction details that were followed were as reported by Komiyama [11]. The quantity was measured in duplicates for each sample. The bacterial copy number was estimated based on the cycle threshold values and standard curves of each sample. Log conversion was performed based on the bacterial load in each picogram of DNA.

### 2.3 Droplet digital PCR (DD-PCR)

DD-PCR was performed using a 16S rDNA detection kit (Targeting One, Beijing, China) and TD-1 Droplet Digital PCR system (Targeting One) according to the manufacturer's instructions. Briefly, a 30 μL dPCR mixture was prepared with 15 μL DNA and 15 μL real-time PCR mix. Then, 30 μL digital PCR mixture and 180 μL oil were loaded onto the droplet generation chip to produce droplets on a drop maker. The droplets were thermally cycled using a protocol of 55 °C for 15 min and 95 °C for 10 min, followed by 40 cycles of 94 °C for 30 s and 57 °C for 1 min. Finally, the droplets were detected using a chip reader (Targeting One). All experiments were performed in duplicates.

### 2.4 Fluorescence in situ hybridisation

Paraffin-embedded tissue samples were dewaxed, incubated with lysozyme, digested with protease K, and hybridised. In the hybridisation buffer, the probe EUB338 (5′-GCTGCCTCCCGTAGGAGT-3′) was added at a dilution ratio of 1:50, and Cy3 red fluorescence was used to mark the gene fragment at the 5′ end of the probe in advance, which was the hybridisation solution. The hybridisation droplets were added to the tissues, covered with a cover glass, and placed in a wet box to prevent the hybridisation liquid from drying overnight. Thereafter, hybridisation was conducted at 42 °C overnight, to avoid light. After hybridisation, washing, 4′, 6-diamidino-2-phenylindole staining, rapid microscopic examination and photography was performed.

### 2.5 16S rDNA high-throughput sequencing and bioinformatics analysis

DNA extraction and high-throughput sequencing were performed by Shanghai Oui Biomedical Technology Co., Ltd. DNA was extracted using MagNA Pure LC 2.0 system and a MagNA Pure LC Total NA Isolation kit (Roche, Mannheim, Baden-Württemberg Land, Germany) in accordance with the manufacturer's instructions and quantified using a Quant-iT PicoGreen dsDNA assay kit (Invitrogen, Eugene, OR, USA). The obtained reads were trimmed using the Sickle software (version 1.33). SAPAdes (version 3.7.1) was used for the hybrid metagenome assembly. The sequencing reads were processed using QIIME (version 1.9.0) [17], and an index of alpha diversity, including Chao 1, PD whole tree, Goods coverage, and observed species, was calculated using QIIME based on a sequence similarity of 97 % (operational taxonomic units, OTU). The distance matrix obtained using UniFrac analysis was used for a variety of analytical methods, and the similarities and differences in microbial evolution in different samples were visualised through principal component analysis (PCA) and non-metric multidimensional scaling of multivariate statistical methods [18]. Differentially abundant taxa were visualised using heat maps with log2-transformed relative abundances and were hierarchically clustered based on the Bray–Curtis distance [19]. To further explore the key genera that may have contributed to the observed differences in the microbial communities, linear discriminant and effect size (LEfSe) analyses [18] were performed to estimate the effect of differentially abundant features with biological consistency and statistical significance.

### 2.6 Statistical analysis

The clinical data of the patients were analysed using Graphpad Prism 8.0.2. Discrete data are represented by the median (range), continuous data are represented by quartile spacing depending on whether they followed normality, and counting data are represented using the Fisher's exact probability method. All statistical tests were two-tailed, and *p* < 0.05 was considered statistically significant.

## 3 Results

### 3.1 Patient information

Among the 16 children with a median age of 32.5 (12–124) months, the number of patients aged <12 months, 12–36 months, and >36 months were 4 (25.0 %), 6 (37.5 %), and 6 (37.5 %), respectively. The participants included 13 boys and three girls. Among the participants, 12 had primary HB and four had recurrent HB. In addition, tumour metastasis was present in seven patients and absent in nine (Table 1). None of the patients were treated with antibiotics within the preceding 15 days.

**Table 1:** Clinical characteristics of patients with hepatectomy [n = 16].

No.	Sex	Age [Months]	Tumor Size [cm]	Tumor Site	Primary or Recurrence	Metastasis	AFP [ng/mL]	ALT [U/L]	AST [U/L]	Tbil [μmol/L]	Dbil [μmol/L]	ALB [g/L]
1	M	24	11 × 7	Right hemi-liver	P	M	60500	8.1	25	6.05	2.17	36.8
2	M	24	7.5 × 7	Left lateral lobe	P	N	65650	8.7	18.8	5.42	2.73	32.8
3	F	48	1.7 × 1.5	Left lateral lobe	R	M	6240	10	23.5	3.8	1.84	42.2
4	M	84	4.5 × 4.5	Right hemi-liver	P	M	11600	35.8	32.3	1.66	1.36	44.2
5	M	48	7.5 × 5	Left hemi-liver	P	N	4488	23.7	39.1	3.63	1.66	43.1
6	M	24	7 × 6	Right hemi-liver	P	N	2088	16.7	36.8	2.03	0.95	44
7	F	36	10 × 10	Right tri-liver	P	N	99.9	758.3	31.4	4.95	1.99	42.6
8	M	124	8 × 7	Right tri-liver	R	M	8429	17.6	26.4	7.98	3.77	39.7
9	M	12	6 × 4	Right tri-liver	P	N	7155	17.6	39.3	2.03	0.75	49.9
10	M	12	10 × 5	Right tri-liver	P	N	72153	17.7	26.8	2.06	1.15	45.4
11	M	84	7 × 4.5	Right tri-liver	R	M	875	13.1	15.7	9.52	3.12	39
12	M	12	12 × 10	Whole liver	P	N	107	5	18.2	7.63	2.78	40.7
13	F	31	7.5 × 6.5	Right tri-liver	P	M	123715	52.8	63.2	3.55	1.08	43.6
14	M	12	7.5 × 7.5	Right tri-liver	P	N	2824	10.9	49.5	7.53	2.72	52.1
15	M	34	11 × 8	Right tri-liver	P	M	36696	26.6	32.5	8.35	2.94	45.6
16	M	72	12 × 10	Left hemi-liver	R	N	665	34.1	24	1.34	0.87	40.5

Note: M, Male; F, Female; P, Primary; R, Recurrence; M, Metastasis; N, No-metastasis; AFP, Alpha fetoprotein, 0–25 ng/mL; ALT, Alanine transaminase, 0-40.

U/L; AST, Aspartate aminotransferase, 0–40U/L; Tbil, Total bilirubin, 5.13–22.24 μmol/L; Dbil, Direct bilirubin,0–6.8 μmol/L; ALB, Albumin, 22–44 g/L.

### 3.2 The core microbiome of the tumour and non-tumour regions in patients with HB

**Fig. 1:**
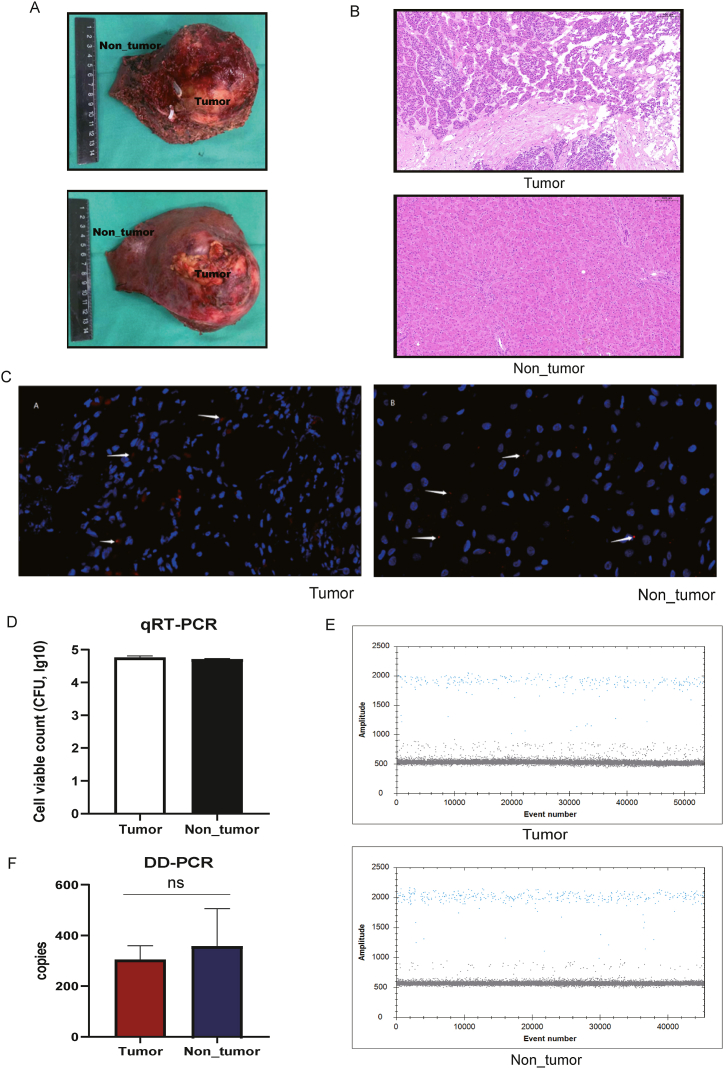
Presence of the microbiome in HB tumours. (A) The HB tumour with surgical resection**.** includes the location of the tumour and adjacent non-tumour tissues. (B) Haematoxylin and eosin staining for HB tumour and adjacent non-tumour tissues. (C) FISH for the confirmation of bacteria in HB tumour and adjacent non-tumour tissues. The red points are labelled bacteria, and the blue nuclei are hepatocytes. The microscope was magnificationx40. (D) Real-time quantitative polymerase chain reaction for detecting the bacteria number in tumour and adjacent non-tumour tissues. (E, F) DD-PCR for detecting the bacteria number in tumour and adjacent non-tumour tissues.

In this study, we obtained tumour tissues from patients with HB during surgical resection to investigate the microbial signatures of HB. We analysed the tumoural and adjacent non-tumoural regions more than 3 cm away from the tumour regions (Fig. 1A). Based on the histopathological results, we were able to distinguish between tumour and non-tumour tissues (Fig. 1B). Fluorescence in situ hybridization confirmed the presence of bacterial DNA in the tumour tissues of patients with HB and their adjacent non-tumour tissues (Fig. 1C). Real-time quantitative polymerase chain reaction revealed no significant differences in bacterial numbers between tumour and adjacent non-tumoural regions. As shown in Fig. 1D, the number of bacteria in the tumour tissues was 4.71 ± 0.14 (log) and that in the adjacent non-tumour tissues was 4.60 ± 0.23 (log) (*p* = 0.056). A similar result was obtained through DD-PCR, confirming the bacterial numbers in the two regions tested (Fig. 1E). Although the average gene expression of 16S rDNA was 304.9 ± 47.7 copies in the tumour region (tumour group) and 354.3 ± 116.8 copies in their adjacent normal tissue regions (non-tumour group), no statistically significant difference was found between them (Fig. 1F).

### 3.3 Romboutsia used as a potential microbial marker to distinguish HB tumour

**Fig. 2:**
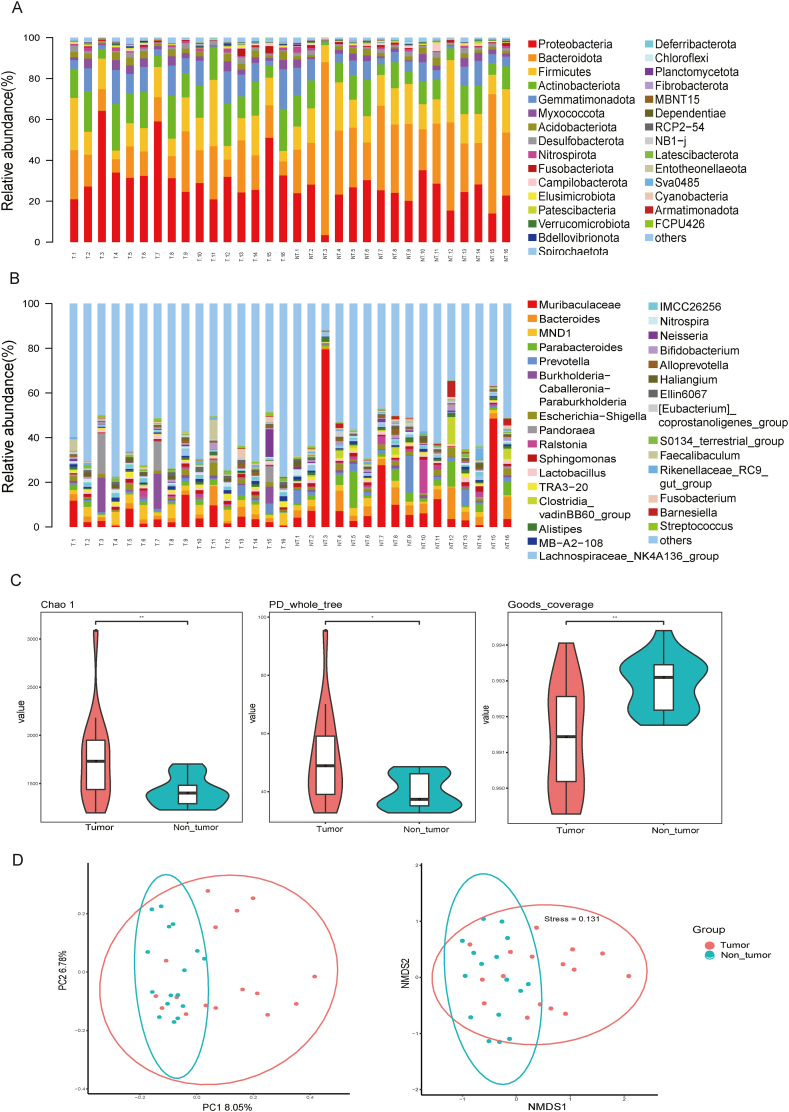
Distribution of the microbiome in HB tumours. (A, B) The bacterial composition of the microbiota at the phylum and genus levels in the tumour and non-tumour regions of HB. Each bar represents an individual sample. (C) Alpha-diversity index, including Chao 1, PD whole tree, and Goods coverage. (D) Principal component analysis of unweighted UniFrac distances among the microbiota in the non-tumour and tumour regions of HB.

Using 16S rDNA sequencing, we detected 35 phyla, 86 classes, 212 orders, 339 families, and 615 genera in 32 tissue samples. The top 10 most abundant phyla were Proteobacteria, Bacteroidota, Firmicutes, Actinobacteriota, Gemmatimonadota, Myxococcota, Acidobacteriota, Desulfobacterota, Nitrospirota, and Fusobacteriota (Fig. 2A). The proportions of Proteobacteria, Bacteroidota, and Firmicutes in the tumour and adjacent non-tumour tissue samples were 33.71 % and 23.42 %, 15.19 % and 33.98 %, and 13.56 % and 15.74 %, respectively. At the genus level, *Muribaculaceae* and *Bacteroides* were dominant (Fig. 2B). The alpha-diversity index values, including Choa 1, PD whole tree, and Goods coverage, were signiﬁcantly different between the tumour and non-tumour groups (*p* < 0.05), indicating that the diversity of the microbiome in the tumour group was signiﬁcantly higher than that in the non-tumour group (Fig. 2C). Using a PCA with the Bray–Curtis distances, the distribution of the microbiome in the tumour group was found to be signiﬁcantly diﬀerent from that in the non-tumour group (*p* < 0.01). This result indicated that the patients had a speciﬁc microbiome profile in their tumour samples (Fig. 2D).

**Fig. 3:**
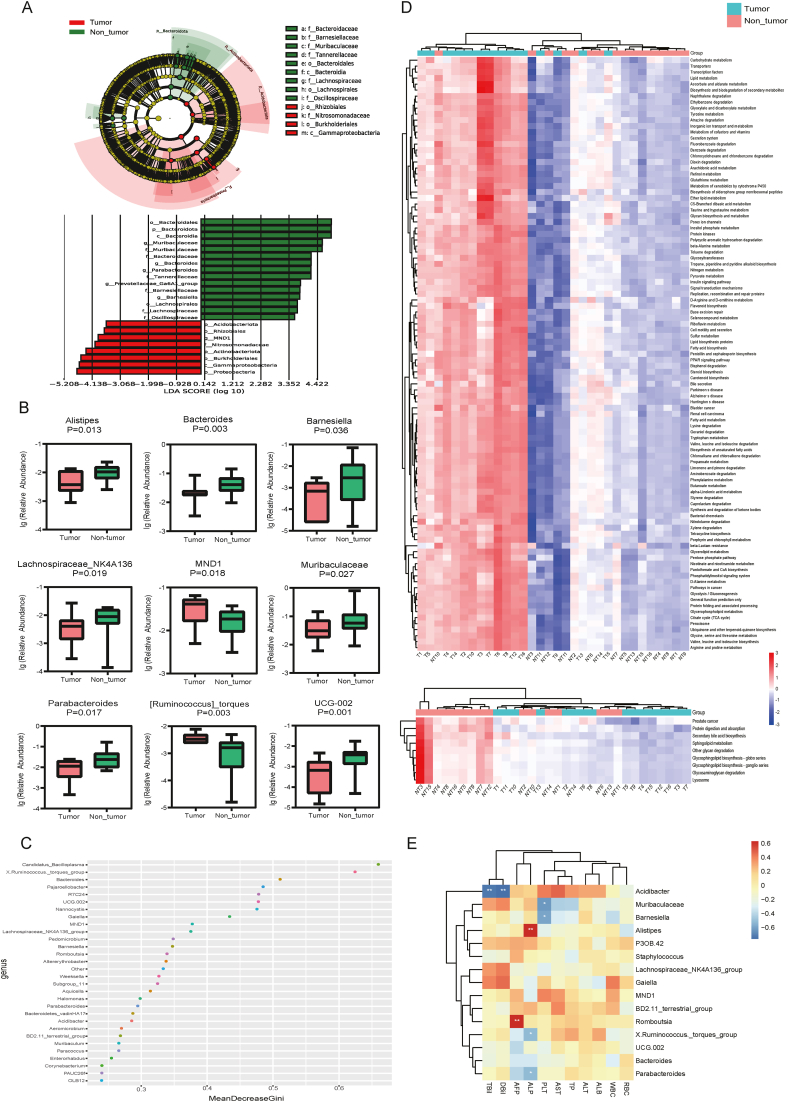
Distinct microbial compositions between tumour and non-tumour tissues of HB. (A) Differences in taxa between the tumour and non-tumour regions of HB determined via the LefSe analysis. (B) Boxplot analysis of the relative abundance of the top nine different genera between the tumour and non-tumour groups. (C) Random forest point map of the top 30 different bacteria genera. (D) Heatmap of the different Kyoto Encyclopaedia of Genes and Genomes pathways for the tumour and non-tumour groups. (E) Heatmap of the association analysis between the differential microbiome and clinical indicators. The red points represent positive correlation, and the bluerepresent negative correlation; ∗ represents *P* < 0.05; ∗∗ represents *P* < 0.01.

LEfSe analysis showed that *Acidobacteriota*, *Nitrosomonadaceae*, *Rhizobiales*, *Actinobacteria*, *Burkholderiales*, *Gammaproteobacteria*, MND1, and *Proteobacteria* were more abundant in the tumour than in the adjacent non-tumour group (Fig. 3A). Using the Wilcoxon rank-sum test, we screened 82 genera that differentiated between the tumour and adjacent non-tumour groups. The nine most abundant genera are presented in Fig. 3B. The abundance of MND1 and *Ruminococcus* in tumour samples was significantly higher than that in adjacent non-tumour samples. *Alistipes*, *Bacteroides*, *Parabacteroides*, *Barnesiella*, *Lachnospiraceaes*-NK4A136, *Muribaculaceae*, *Parabacteroids*, and UCG-002 were significantly less abundant in the tumour than in the adjacent non-tumour group. To further screen for the more important microbiome, which plays an important role in the progression of tumours, we used a random forest to rank the top 30 bacteria with statistically significant differences between the two groups (*p* < 0.01), as shown in Fig. 3C. In addition, the Kyoto Encyclopaedia of Genes and Genomes (KEGG) pathway enrichment analysis was conducted to gain insights into the functional changes in tumour microbiomes in patients with HB. Compared with those of the samples of the non-tumour group, the KEGG pathways of most samples (10/16) of the tumour group were enriched and highly expressed in the circulatory system, neurodegenerative diseases, cell motility, xenobiotics biodegradation and metabolism, signal transduction, energy metabolism, genetic information processing, carbohydrate metabolism, cancers, etc. Moreover, sphingolipid metabolism was decreased in the tumour group (Fig. 3D). To understand the relationship between the microbiome in HB tissues and the clinical indicators of disease, Spearman's correlation analysis was conducted between the 15 bacteria and clinical indicators (Fig. 3E). The microbiome in HB tissues significantly correlated with the main clinical indicators. In particular, *Romboutsia* strongly correlated with alpha-fetoprotein, an important indicator of HB, suggesting that *Romboutsia* can be used as a potential microbial marker to distinguish HB tumour tissues.

### 3.4 Distinct microbial compositions between primary and recurrent HB

**Fig. 4:**
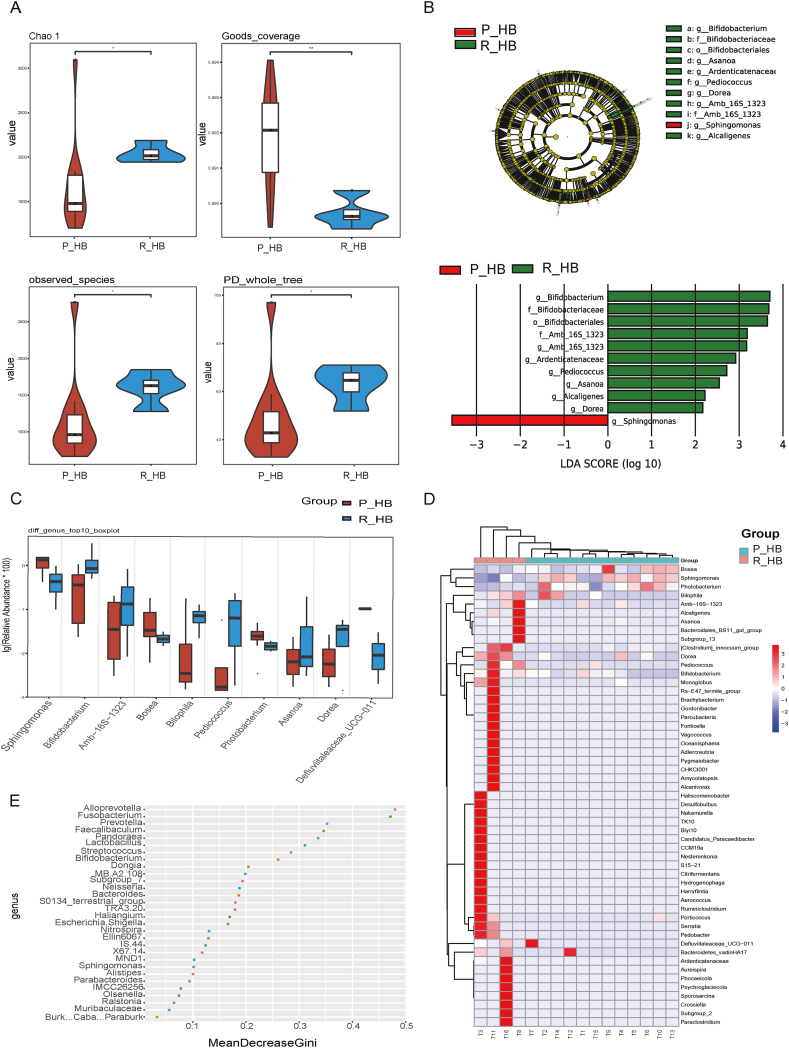
Distinct microbial compositions between primary HB (P_HB) and recurrent HB (R_HB). (A) Alpha-diversity index, including the Chao 1, PD whole tree, Goods coverage, and observed species. (B) Differences in taxa between the P_HB and R_HB groups determined via the LefSe analysis. (C) Boxplot analysis of the relative abundance of the top nine different genera between the P_HB and R_HB groups. (D) Heatmap of the microbiome profile for each sample in the P_HB and R_HB groups. (E) Random forest point map of the top 30 different bacteria genera.

Among the 16 patients, four belonged to the recurrent HB group. To assess whether the microbiome was associated with tumour recurrence, we compared the alpha- and beta-diversity indices of the microbiome between the primary HB (P_HB) and recurrent HB (R_HB) groups. As shown in Fig. 4A, the index values, including Chao 1, PD whole tree, Goods coverage, and observed species were signiﬁcantly different between the P_HB and R_HB groups (*p* < 0.05). LEfSe analysis results showed that in the R_HB group, *Bifidobacterium*, Amb_16S_1323, *Ardenticatenaceae*, *Pediococcus*, *Asanoa*, *Alcaligenes*, and *Dorea*, were abundant in the microbiome. *Sphingomonas* was abundant only in the P_HB group (Fig. 4B).

Using the Wilcoxon rank-sum test, we screened the top 10 genera: *Sphingomonas*, *Bifidobacterium*, Amb_16S_1323, *Bosea*, *Bilophila*, *Pediococcus*, *Photobacterium*, *Asanoa*, *Dorea*, and *Defluviitaleaceae*. As shown in Fig. 4C, these genera differed significantly between the P_HB and R_HB groups (*p* < 0.05). We identified the 30 most important bacterial genera on a random forest point map (Fig. 4E). *Sphingomonas* and *Bifidobacterium* were screened through this method, consistent with the screenings by other analytical methods. *Sphingomonas* was more abundant in the P_HB group and *Bifidobacterium* was more abundant in the R_HB group. According to the heatmap of the different samples with respect to the genus (Fig. 4D), every sample in the R_HB group possessed specific microbiome profiles; therefore, the microbiome was disordered when recurrent HB occurred.

### 3.5 Distinct microbial compositions between metastatic and non-metastatic HB

**Fig. 5:**
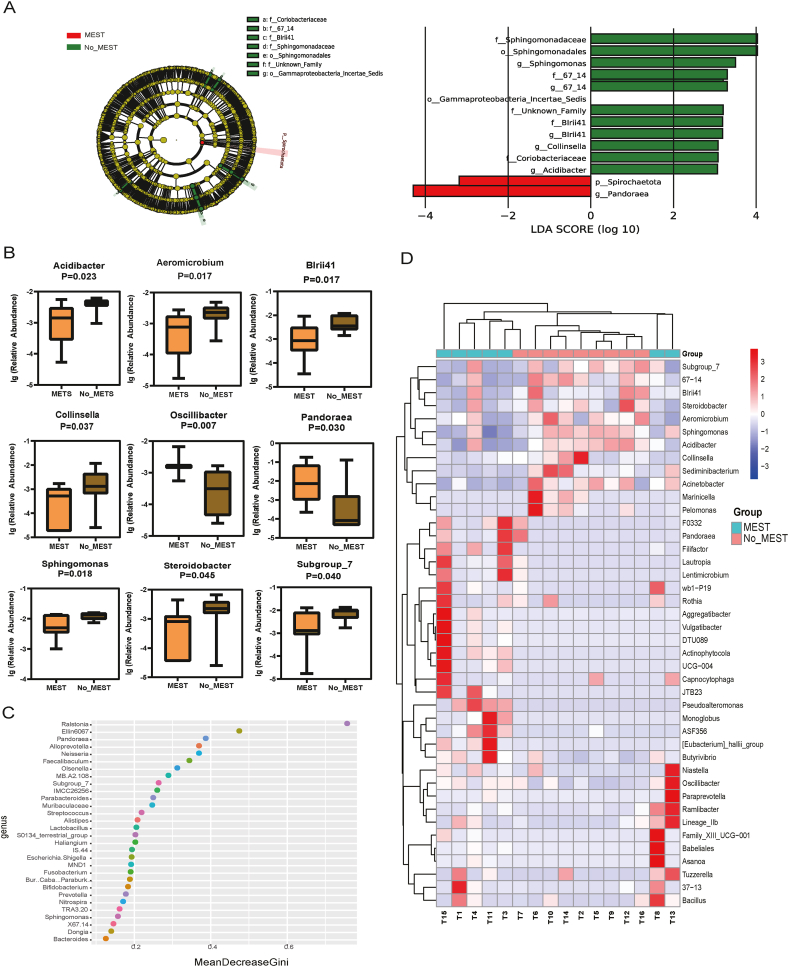
Distinct microbial compositions between metastatic HB (M_HB) and non-metastatic HB (NM_HB) groups. (A) Differences in taxa between the M_HB and NM_HB groups determined via the LefSe analysis. (B) Boxplot analysis of the relative abundance of the top nine different genera between the M_HB and NM_HB groups. (C) Random forest point map of the top 30 different bacteria genera. (D) Heatmap of microbiome profile for each sample in the M_HB and NM_HB groups.

Based on the presence or absence of metastasis, the cases were separated into metastatic HB (M_HB) and non-metastatic HB (NM_HB) groups. LEfSe analysis showed that *Spirochaetota* and *Pandoraea* were more abundant in the M_HB than in the NM_HB group (Fig. 5A). Using the Wilcoxon rank-sum test, we screened the top 9 genera, including *Acidibacter*, *Aeroicrobium*, *BIrii*41, *Collinsella*, *Oscillibacter*, *Pandoraea*, *Sphingomonas*, *Steroidobacter*, and Subgroup7. As shown in Fig. 5B, these genera differed significantly between the M_HB and NM_HB groups (*p* < 0.05). We identified the 30 most important bacterial genera on a random forest point map (Fig. 5C). *Pandoraea*, Subgroup7, and *Sphingomonas* were screened through this method, consistent with the screenings by other analytical methods. The samples in the NM_HB group were clustered according to the heatmap of the different samples by genus (Fig. 5D).

## 4 Discussion

The microbiome regulates and contributes to liver diseases, including non-alcoholic fatty liver disease, cirrhosis, and hepatocellular carcinoma (HCC) [[20], [21], [22]]. The steady-state destruction of the microecosystem between the microbiome and host may in turn lead to the destruction of the intestinal barrier function and migration of bacteria from the intestinal tract to other extraintestinal organs or parts, including the liver [23]. Therefore, with intestinal epithelial barrier dysfunction, an increase in intestinal permeability can be observed in patients with chronic liver disease, indicating that intestinal endotoxins promote the development of liver diseases [16,24]. The intestinal microbiome is an important factor affecting drug metabolism, inflammation, cancer progression, and cancer treatment outcomes [15,25]. However, the mechanism by which the tumour microbiome leads to HB development in children remains unclear. It has been reported that caesarean sections are associated with an increased risk of the birth of children with hepatoblastoma, possibly due to a change in their microbiome [26].

Komiyama et al. found that the tumour-associated microbiome domain in human HCC consists of Bacteroidetes, Firmicutes, and Proteobacteria [11]. Moreover, these three phyla are the main taxa in various tumour tissues, such as bones, breasts, colon, lungs, and ovaries [16,[27], [28], [29]], and are also the main members of the human intestinal microbiome [30]. In addition, they identified an unclassified genus belonging to *Bacteroides*, *Romboutsia*, and an uncultured bacterium of the Lachnospiraceae family as unique microbiomes in primary liver cancer. Another study identified *Ruminococcus gnavus* as a biomarker for the tumour region in patients with viral HCC infected with hepatitis B and/or hepatitis C viruses [11]. Similar to the above results, we found that the domain microbiomes in HB tumours and adjacent normal tissues were Proteobacteria, Bacteroidetes, and Firmicutes. Moreover, *Bacteroides*, *Romboutsia*, *Ruminococcus*, and *Lachnospiraceae* were found to be associated with HB. Compared with those in the adjacent non-tumour tissues, the abundances of *Ruminococcus* and *Romboutsia* were higher in tumour tissues, whereas those of *Bacteroides* and *Lachnospiraceae*-NK4A136 were lower. Notably, this study demonstrated a strong correlation between *Romboutsia* and serum alpha-fetoprotein. In a previous study, *Ruminococcus* was found to induce dendritic cells to produce tumour necrosis factor alpha (TNF-α) by making a complex glucan [31], while TNF-α was found to promote cancer. Therefore, we verified *Ruminococcus* and *Romboutsia* as potential biomarkers of HB. In addition, it is worth considering that compared with adjacent normal tissues, HB tumour tissues lacked *Bacteroides* and *Alistipes*, indicating a certain correlation of these microbiomes with cancer. *Bacteroides* can produce acetic acid, propionic acid, and butyric acid salts as the main end products of sugar fermentation [32]. Acetic acid salts can prevent the transport of toxins between the lumen and blood, whereas propionate can induce apoptosis in human colon cancer cells [33]. Portal vein propionic acid levels help prevent liver cancer cell proliferation. Similarly, *Alistipes* is regarded as an important producer of short-chain fatty acids, and the activity of short-chain fatty acids in fermented dietary fibres regulates the surrounding environment and directly interacts with the host immune system [34]. *Alistipes* can also prevent the proliferation of HCC cells in liver tissue by increasing propionic acid levels in the portal vein [35]. Therefore, the loss of these microbiomes in HB tumour tissues may be related to the occurrence and development of HB.

To assess whether there were representative microbiomes in the recurrent HB tumours, we compared the P_HB and R_HB groups. Although some microbiomes were abundant in recurrent HB tumours, every sample possessed a specific microbiome profile in the heatmap. Therefore, identifying the representative microbiomes of these bacteria was challenging. However, there was an increase in *Sphingomonas* in primary HB tumours, and its abundance was significantly different between the P_HB and R_HB groups. *Sphingomonas*, which affects xenobiotic biodegradation and genomic instability of the host, has been reported to be associated with thyroid carcinoma, thymic epithelial tumours, and bladder cancer [[36], [37], [38]], and this bacterium has potential roles in primary HB. In addition, considering that some patients with metastatic cancer have live bacteria in their tumour tissues, detecting bacterial activity in HB tissues will help in increasing our understanding of the pathogenic role of tumour-associated microbial populations. Therefore, to assess whether there were representative microbiomes were present in metastatic HB, we compared the M_HB and NM_HB groups. Among the nine genera that differed between the M_HB and NM_HB groups, *Oscillibacter* and *Pandoraea* were abundant rich in metastatic HB. Some studies have found that the relative abundance of *Oscillibacter* is remarkably increased in patients with secreting pituitary adenoma (GHPA) and positively correlated with depressive behaviours [39,40]. *Pandoraea* species are multidrug-resistant, glucose-nonfermenting, gram-negative bacilli that are usually isolated from patients with cystic fibrosis [41]. Therefore, the association of these bacteria with metastatic HB tumours requires further investigation. A limitation of this study is the small sample size drawn from a single institution. Because the incidence of HB is 1.2–1.5/10^6^ per year [2], obtaining more samples to identify the characteristics of cancer microbiome is cumbersome. Therefore, further studies on the relationship between the microbiome and HB are required.

In summary, we identified a tumour-associated microbiome in HB. The study findings showed that Bacteroidetes, Firmicutes, and Proteobacteria are the main taxa in HB tumours and are prominent members of the human intestinal microbiome. Therefore, considering the anatomical locations of the liver, portal vein, and intestine, the intra-tumoural microbiome found in the HB is most likely to stem from the intestinal microbiome. *Ruminococcus* and *Romboutsia* were identified as potential biomarkers of HB tumours. Overall, our findings shed light on the microbiome characteristics of HB, which can contribute to our understanding of the mechanisms underlying tumour-associated microbiome development in liver tumours.

## CRediT authorship contribution statement

**Jinghua Cui:** Writing – original draft. **Xiaoran Li:** Writing – original draft, Resources. **Qun Zhang:** Methodology, Formal analysis. **Bing Du:** Software. **Zanbo Ding:** Validation. **Chao Yan:** Methodology. **Guanhua Xue:** Validation. **Lin Gan:** Software. **Junxia Feng:** Methodology. **Zheng Fan:** Validation. **Ziying Xu:** Data curation. **Zihui Yu:** Formal analysis. **Tongtong Fu:** Data curation. **Yanling Feng:** Resources. **Hanqing Zhao:** Resources. **Yiming Kong:** Resources. **Xiaohu Cui:** Methodology. **Ziyan Tian:** Software. **Quanda Liu:** Writing – review & editing, Conceptualization. **Jing Yuan:** Writing – review & editing, Project administration.

## Ethics approval and consent to participate

This study was approved by the Ethics Committee of Rocket Force Special Medical Center (KY2024040) and followed the ethical principles of the Declaration of Helsinki. All the children had their surgical informed consent obtained by their families.

## Data availability and supplementary material

The datasets presented in this study can be found in online repositories. The names of the repository/repositories and accession number(s) can be found below: https://bigd.big.ac.cn/gsa/browse/CRA009419.

## Funding

This work was supported by grants from the 10.13039/501100001809National Natural Science Foundation of China (82272352), Research Foundation of Capital Institute of Pediatrics (JHYJ-2023-02), Beijing Municipal Public Welfare Development and Reform Pilot Project for Medical Research Institutes (JYY2023-19), Beijing High-Level Public Health Technical Talent Project (2023-02-08). The experiments conducted in this study comply with the current laws of the countries in which they were performed.

## Declaration of Competing interest

All authors declare that No conflict of interest exists.
